# Assessment of Subclinical Psychotic Symptoms in Patients with Rheumatoid Arthritis and Spondyloarthritis

**DOI:** 10.3390/jcm10163461

**Published:** 2021-08-04

**Authors:** Juan L. Prados-Ojeda, Rogelio Luque-Luque, Rafael M. Gordillo-Urbano, Ipek Guler, Clementina López-Medina, Eduardo Collantes-Estévez, Alejandro Escudero-Contreras

**Affiliations:** 1 Mental Health Department, Reina Sofia University Hospital, 14004 Cordoba, Spain; juanlprados@gmail.com (J.L.P.-O.); rogelioluque@uco.es (R.L.-L.); rmgordillo32@gmail.com (R.M.G.-U.); 2 Morphological and Socio-Sanitary Sciences, University of Cordoba, 14004 Cordoba, Spain; 3 Maimonides Institute of Biomedical Research of Córdoba (IMIBIC), 14004 Cordoba, Spain; ipek.guler@kuleuven.be (I.G.); educollantes@yahoo.es (E.C.-E.); alexcudero2@gmail.com (A.E.-C.); 4 Medical and Surgical Sciences Department, University of Cordoba, 14004 Cordoba, Spain; 5 Rheumatology Department, Reina Sofia University Hospital, 14004 Cordoba, Spain

**Keywords:** rheumatoid arthritis, spondyloarthritis, inflammation, autoimmunity, psychotic symptoms, anxiety, depression

## Abstract

Inflammatory and autoimmune processes have been associated with the onset of depressive and psychotic symptoms. Rheumatoid arthritis (RA) and spondyloarthritis (SpA) are rheumatic diseases with an inflammatory etiology. A high prevalence of depressive and anxiety-related comorbidity has been reported for both diseases, with no evidence of a greater prevalence of psychosis. The objective of the present study was to evaluate for the first time subclinical psychotic symptoms in patients with RA and SpA. This is a cross-sectional, single-center study including RA and SpA patients, as well as healthy controls. Abnormal psychotic experiences (positive, negative, and depressive symptoms) were evaluated using the Community Assessment of Psychic Experiences (CAPE-42). Functional capacity was evaluated using the Short-Form Health Survey SF-12. We compared the CAPE and SF-12 scores between the three groups. We recruited 385 individuals: 218 with RA, 100 with SpA, and 67 healthy controls. According to the CAPE scale, the frequency of subclinical psychotic symptoms was greater in patients than in healthy controls (RA, 1.90 vs. 1.63, *p* < 0.001; SpA, 1.88 vs. 1.63, *p* = 0.001). Distress was also greater in patients than in controls owing to the presence of symptoms. No differences were observed between the three groups for the mental dimension scores in the SF-12 Health Survey (43.75 in RA, 45.54 in SpA, and 43.19 in healthy controls). Our findings point to a greater prevalence of subclinical psychotic symptoms in patients with RA and patients with SpA than in the general population. The results suggest an association between inflammation and depression/subclinical psychotic symptoms.

## 1. Introduction

Recent studies have shown the role of inflammatory mechanisms [1,2,3,4] and autoimmune mechanisms in the onset of psychiatric symptoms, such as depression [5,6,7]. In this sense, structural similarities have been identified between anti-inflammatory drugs and some antidepressants [8].

In addition to their possible role in depression, inflammatory processes have been associated with the onset of psychotic symptoms [9,10]. Thus, inflammation has been reported to play a role in initial episodes of psychosis [11,12], with evidence for the presence of peripheral inflammatory markers (e.g., C-reactive protein) [13,14] during psychotic episodes, together with the activation of microglia [15,16]. As in the case of antidepressants, the anti-inflammatory capacity of antipsychotics has also been evaluated [17], and some studies have examined the role of anti-inflammatory drugs as a new tool for the treatment of psychosis [18].

More recent reports have shown evidence for an association between the onset of psychosis and some autoimmune processes [19]. Autoimmune activity has been observed in acute psychotic episodes owing to the presence of anti-N-methyl-D-aspartate autoantibodies, anti-basal ganglia autoantibodies, and other autoantibodies [20]. Similarly, genetic studies have highlighted the importance of the major histocompatibility complex and its relationship with schizophrenia [21].

Rheumatoid arthritis (RA) and spondyloarthritis (SpA) are two of the most common inflammatory rheumatic diseases in the general population [22], with a prevalence as high as 1%. Inflammatory processes play a role in the etiology of both diseases. In RA in particular, an underlying autoimmune process has been observed, together with characteristic antibodies. In SpA, on the other hand, inflammation is the predominant process.

There is an extensive body of literature on the high prevalence of psychiatric comorbidity, mainly depression and anxiety, in both diseases [23,24,25,26,27,28,29]. However, to date, no publications have reflected a greater prevalence of psychotic episodes for either of these diseases. In fact, in the case of RA, an inverse association has been reported between RA and the most representative manifestation of psychosis, namely, schizophrenia [30,31,32,33]. Similarly, a recent study reported this same negative association not only for RA, but also for SpA [19].

Psychiatric research has given rise to many studies on the presence of subclinical psychotic symptoms in the general population. These report a greater prevalence than expected of such symptoms in persons without psychiatric disorders [34,35,36,37]. While these symptoms are not sufficiently severe to require medical attention, they have been associated with the level of functioning and quality of life in persons who experience them [38]. Nevertheless, to date, subclinical psychotic symptoms have not been evaluated in patients with RA and SpA, in whom prevalence may be increased owing to the inflammatory etiology and possibly also to the role of autoimmune processes.

The objective of the present study was to evaluate for the first time the presence of subclinical psychotic symptoms in the two most representative rheumatic diseases, i.e., RA and SpA (each of which has different etiological–pathogenic mechanisms), and to compare affected patients with healthy controls.

## 2. Materials and Methods

### 2.1. Study Design

We performed a single-center, cross-sectional study in the Departments of Rheumatology and Mental Health at Hospital Universitario Reina Sofía, Córdoba, Spain. 

The study was approved by the Research and Ethics committee of Hospital Universitario Reina Sofía, Córdoba (22 December 2016. Ref. 3371).

### 2.2. Participants

The study population comprised patients aged ≥18 years diagnosed with RA or SpA and followed by a Rheumatologist at least during the 12 moths prior to the study. Patients were recruited through various channels: (a) Department of Rheumatology, Hospital Universitario de Reina Sofía, Córdoba, Spain; (b) Asociación Cordobesa de Enfermos de Artritis Reumatoide (ACOARE (Cordoba Association of Patients with Rheumatoid Arthritis)); (c) Asociación Cordobesa de Enfermos Afectados de Espondilitis (ACEADE (Cordoba Association of Patients with Spondylitis)); and (d) an advertisement on the webpage of the Coordinación Nacional de Artritis (CONARTRITIS (National Arthritis Coordination Association)). Patients included from outpatient consultation and those who completed the online survey were enrolled consecutively.

The control group comprised individuals who did not have rheumatic disease (mainly members of the patients’ immediate families) who responded to the surveys voluntarily via the associations and webpage, as well as the persons accompanying patients who were attending the hospital. 

The exclusion criteria were age <18 years, diagnosis of a rheumatic disease other than RA or SpA, or absence of follow-up by a Rheumatologist.

All those invited to participate in the present study were provided with documentation and complete information regarding the study prior to providing their voluntary written informed consent to participate.

### 2.3. Variables and Data Sources

Study data were obtained using a paper document or an online questionnaire. The variables recorded were as follows:

- Sociodemographic data: age, sex, and marital status.
- Clinical data: diagnosis (RA, SpA, healthy). Data were also collected on current consumption of alcohol and other drugs, smoking, previous drug consumption, and psychiatric history (understood as a previous consultation with a mental health professional or prescription of psychiatric drugs).
- Measurements:
  ○ Abnormal psychotic experiences: We used the validated Spanish version [39] of the Community Assessment of Psychic Experiences (CAPE-42) [40], a self-administered questionnaire with 42 questions that evaluates the frequency of presentation of three groups of symptoms, as follows: positive symptoms, i.e., those associated with the presence of perceptual anomalies, bizarre experiences, and delusional ideas (20 items); negative symptoms, such as affective flattening, avolition, and anhedonia (14 items); and depressive symptoms (8 items), as well as the degree of distress experienced by the individual owing to the presence of these symptoms. The scale used comprises four points both for frequency (never, sometimes, often, and nearly always) and for distress (not distressed, a bit distressed, quite distressed, and very distressed).
  ○ The Short Form Health Survey SF-12. SF-12 is a self-administered scale comprising 12 items. The original version comprises 36 items (SF-36). SF-36 was used to develop a shorter version that has proven to be a useful alternative (SF-12) [41]. SF-12 is a subset of 12 items, including 1 or 2 items from each of the 8 scales of SF-36. We used the validated Spanish version [42] in the present study. The scale is widely used to evaluate the patient’s functional status and makes it possible to evaluate health-related quality of life. The questionnaire comprises two dimensions by which physical and mental health are evaluated.

### 2.4. Statistical Analysis

The sample size was estimated considering an alpha risk of 5% and a power of 80% to detect a clinically relevant difference in CAPE total frequency of 0.15, with a standard deviation (SD) of 0.30. According to the hypothesis that a CAPE total frequency in the control group will be 1.50 and a 2:1 ratio, the minimum estimated sample size was 64 healthy controls, 128 SpA, and 128 RA.

We collected the mean CAPE scores, both overall and for each group of symptoms (positive, negative, and depressive), as well as the mean scores for frequency and distress. 

We conducted a descriptive analysis comparing mean CAPE scores for each symptom group among the three groups of participants (RA, SpA, and healthy) using one-way analysis of variance (ANOVA) and the Tukey–Kramer test for post hoc pairwise comparisons. *p*-values less than 0.05 were considered to indicate statistical significance. Next, we used the chi-square test to evaluate each CAPE dimension across the three groups and the Mann–Whitney test to explore differences in the CAPE scores between the groups.

Finally, a multivariate linear regression analysis was performed to evaluate factors independently associated with SF-12, using age, sex, marital status, smoking, alcohol, and drug use as covariates.

The analysis was performed using the free software application R (version 3.5.0).

## 3. Results

### 3.1. Characteristics of the Population

The sociodemographic data of the participants are shown in Table 1. We recruited 385 individuals aged 18–85 years (mean, 46.33 [12.19] years). Most patients had RA (218, 56.6%), 100 had SpA (25.9%), and 67 were healthy (17.4%). We found differences between the groups for sex, with significant differences when RA and SpA patients were compared with healthy controls (*p* < 0.001). A psychiatric history was recorded for 73.4% of RA patients, 75% of SpA patients, and 50.7% of healthy controls, with statistically significant differences between the groups. As for substance use, we found no differences regarding smoking or consumption of other drugs, although alcohol was consumed more frequently by healthy individuals than by patients with SpA and RA (16.4% vs. 11% vs. 5%) (*p* = 0.009). Numerical—but not significant—differences were recorded for marital status, where being single or separated was more frequent in the RA group (34.8%) than in the SpA group (24%) or control group (25.3%).

### 3.2. Mean CAPE-42 Scores

Significant differences in the CAPE-42 scores were found between the groups regarding the total score in the frequency of presentation of symptoms and subsequent distress (*p* < 0.001) (Table 2).

The total frequency score was greater for RA than for healthy controls (1.68 vs. 1.49, *p* < 0.001) and greater for SpA than for healthy controls (1.65 vs. 1.49, *p* = 0.002). Furthermore, significant differences were observed for the total distress score between RA and healthy controls (2.40 vs. 2.03, *p* < 0.001) and between SpA and healthy controls (2.28 vs. 2.03, *p* = 0.009). No significant differences in total score were found when the RA and SpA groups were compared.

We also found statistically significant differences (*p* < 0.001) in the subscales for frequency of negative and depressive symptoms. Differences were found in the frequency of negative symptoms between RA and healthy controls (1.90 vs. 1.63, *p* < 0.001) and between SpA and healthy controls (1.88 vs. 1.63, *p* = 0.001). As for the frequency of depressive symptoms, differences were found between RA and healthy controls (2.07 vs. 1.63, *p* < 0.001) and between SpA and healthy controls (2.04 vs. 1.73, *p* < 0.001). No significant differences were found in the frequency of positive symptoms in any of the diseases compared with controls.

Analysis of the distress subscales revealed significant differences for all the groups and for the three types of symptoms (positive and depressive symptoms (*p* = 0.001) and negative symptoms (*p* < 0.001)). In the case of distress generated by positive symptoms for RA and healthy controls, we found that mean distress was greater in the former (2.05 vs. 1.71, *p* < 0.001); the findings were similar when SpA patients were compared with healthy controls (1.94 vs. 1.71, *p* = 0.026). As for distress caused by negative symptoms, differences between patients with the diseases and healthy controls were found for RA (2.45 vs. 2.04, *p* < 0.001) and for SpA (2.31 vs. 2.04, *p* = 0.008). In the case of distress caused by depressive symptoms, we only found significant differences when patients with RA were compared with healthy controls (2.66 vs. 2.29, *p* = 0.001). No significant differences in the subscales were found on comparing RA and SpA.

As for prevalence of the symptoms in the three groups (taking into account only the responses “often” and “nearly always”, which imply greater frequency of symptoms), we observed prevalence rates of positive symptoms of 53% in RA patients, 53% in SpA patients, and 34% in healthy controls. As for negative symptoms, we observed prevalence rates of 71% in RA patients, 73% in SpA patients, and 48% in healthy controls. Lastly, the prevalence rates of depressive symptoms were 63% in RA, 58% in SpA, and 41% in healthy controls.

### 3.3. Mean SF-12 Scores

The results for the SF-12 are shown in Table 2. We observed significant differences in the physical dimension scores after comparing both RA patients (32.58) and SpA patients (30.02) with healthy controls (53.31) (*p* < 0.001).

No differences were observed for the scores on the mental dimension, which were very similar in all the groups: RA, 43.75; SpA, 45.54; and healthy controls, 43.19.

### 3.4. Multivariate Analysis of SF-12 Scores

We performed a multivariate analysis (Table 3) on the SF-12 scores. The results showed that the mental dimension was independently and positively associated with SpA (*p* = 0.023) and age (*p* < 0.001), whereas a negative correlation was observed for psychiatric history (*p* < 0.001) and smoking (*p* = 0.001). In the physical dimension, the association was independent and negative for diagnosis of RA and SpA (*p* < 0.001), age (*p* = 0.002), sex (*p* = 0.024), and psychiatric history (*p* = 0.001).

## 4. Discussion

Our findings suggest that the prevalence of subclinical psychotic symptoms is greater in patients with RA or SpA than in the general population, thus highlighting the presence of a comorbid condition that has received little attention in these patients.

The high prevalence of anxiety and depression is well documented in patients with RA [23,24,25,26] and in patients with SpA [27,28,29], thus pointing to a possible association between inflammatory processes and the etiology and pathogenesis of depression [1,2,3,4]. Our study confirms the association between these two inflammatory diseases and both depression and subclinical psychotic symptoms, as we found a greater prevalence of these symptoms in patients with RA and SpA than in healthy controls. However, our results contrast with those of previous studies, which showed scant psychotic comorbidity or even an inverse relationship [19,30,31,32,33] between psychotic symptoms and both diseases.

Current hypotheses on the etiology of psychotic symptoms discuss the role of interleukins [43,44], tumor necrosis factor [45], autoantibodies [20], and factors associated with oxidative stress [12,46,47], all of which are also involved in RA and SpA. Based on this information and given that, within the field of psychiatric research, many studies performed in the nonpsychotic general population report subclinical psychotic symptoms [35,36,37], it seems relevant to investigate the presence of these symptoms in patients with RA and SpA.

The differences we found are particularly interesting regarding the frequency of negative and depressive symptoms. Negative symptoms have been associated with inflammatory mechanisms [48] and include avolition and affective flattening, with the involvement of reward mechanisms [9]. Negative symptoms have been associated with a lower level of social functioning [49] and poorer cognitive performance [50], both of which are widely cited as part of the clinical picture during the progression of RA [51,52] and SpA [53,54]. Therefore, the presence of negative subclinical symptoms gains in importance owing to the effect on both social and cognitive functioning, the involvement of which also affects quality of life [38] and disability [55]. Moreover, in the literature, the presence of subclinical psychotic symptoms has been associated with the onset of depressive symptoms [56,57]. Therefore, the highly prevalent negative symptoms detected in the RA and SpA groups (including abulia and affective flattening) are easily identified as depressive symptoms, which could mistakenly increase the reported prevalence of depression in these patients.

Although CAPE scores were higher in both diseases in comparison with healthy controls, no differences were found in the SF12 mental dimension between groups. This could be explained by the subclinical nature of PLEs, which are not identified by the patients as a psychiatric symptom requiring clinical attention. In this sense, the subclinical nature of PLEs could also explain the positive correlation between SpA patients and SF12 mental dimension, while CAPE scores were no different between SpA and RA. As expected, smoking and psychiatric history correlated negatively with the SF12 mental dimension.

Mechanisms of autoimmunity [19,20,21] and inflammation [9,10,13,14,15,16] have been reported to play a role in the etiology of psychotic disorders. In our study, we included a sample of patients with RA, in which both autoimmunity and inflammation play a key role, and patients with SpA, in which inflammation is the basic etiological–pathogenic mechanism, without the involvement of immunological factors. Our data revealed no differences in the presence of subclinical psychotic symptoms between the two diseases, indicating that inflammation could be the main mechanism involved in the presence of symptoms. New studies involving patients with inflammatory and autoimmune diseases, such as those recruited in our sample, will be necessary before this potential association can be confirmed.

Given that the presence of negative symptoms has been associated with a lower level of functioning that may directly affect not only a patient’s quality of life, but also aspects such as adherence and response to treatment [23,58], it is important to detect the disease in early stages of RA. Various psychotherapeutic and psychosocial interventions could prove useful for negative symptoms [59,60]. Undoubtedly, early use of these tools could play a role in patients’ coping strategies and improve disease progress.

New studies are necessary to confirm the presence of subclinical psychotic symptoms both in patients with RA and in those with SpA, mainly in the early phases of the disease, and thus confirm our findings.

This study has some limitations. First, the cross-sectional design of this study prevents the establishment of a causal relationship. An association between psychotic symptoms and rheumatic disease can be established from the results of this study, although future longitudinal analysis should be conducted to better understand this relationship. The use of electronic records may lead to a selection bias because women and young people are more likely to participate in electronic surveys. The study is also limited by a possible selection bias regarding the healthy controls that were recruited from the volunteers’ families through the websites of patient associations. Nevertheless, the fact that patients and their controls lived together would probably mitigate this variability. The last limitation is represented by our inability to collect data on disease activity or on the degree of disability at the time of the study owing to the nature of the study.

As for the online and paper responses, some studies suggest that the CAPE-42 scores are slightly lower when the questionnaire is completed online, although these differences have not been shown to be important in research studies [61]. Most of the responses in the present study were made online. However, both methods were used in all of the groups, thus minimizing any possible impact.

In summary, our study is the first to compare the prevalence of subclinical psychotic symptoms in patients with RA and patients with SpA to that in healthy controls. These symptoms should be regarded as comorbid conditions to be taken into account when evaluating the patient.

## Figures and Tables

**Table 1:** Sociodemographic factors of the total population.

	Rheumatoid Arthritis *n* = 218	Spondyloarthritis *n* = 100	Healthy Controls *n* = 67	*p*-Value
Age (years), mean (SD) Median (min–max)	47.35 (12.18) 48 (18–85)	45.34 (12.38) 44 (22–78)	44.51 (11.57) 43 (18–74)	0.106
Sex				
- Male, *n* (%) - Female, *n* (%)	22 (10.09) 196 (89.90)	36 (36) 64 (64)	21 (31.34) 46 (68.65)	<0.001 ^a,c^
Marital Status				
- Married or couple, *n* (%) - Separated or divorced, *n* (%) - Single, *n* (%) - Widowed, *n* (%)	139 (63.76) 24 (11.0) 54 (24.8) 1 (0.46)	75 (75) 3 (3.00) 21 (21.0) 1 (1.00)	50 (74.63) 4 (5.97) 13 (19.4) 0 (0.00)	0.114
Psychiatric history, *n* (%)	160 (73.39)	75 (75)	34 (50.70)	0.001 ^a,b^
Daily users of alcohol, *n* (%)	11 (5.04)	11 (11)	11 (16.41)	0.009 ^a^
Daily smokers, *n* (%)	42 (19.26)	22 (22)	6 (8.95)	0.083
Other drugs, *n* (%)	8 (2.98)	6 (6)	2 (2.98)	0.602
History of drug use, *n* (%)	35 (16.1)	18 (18.0)	12 (17.9)	0.884
Source:				
Internet, *n* (%) Paper, *n* (%)	185 (84.9) 33 (15.1)	90 (90.0) 10 (10.0)	49 (73.1) 18 (26.9)	0.013 ^a,b^

^a^ Post hoc significant differences between rheumatoid arthritis and healthy controls. ^b^ Post hoc significant differences between spondyloarthritis and healthy controls. ^c^ Post hoc significant differences between rheumatoid arthritis and spondyloarthritis.

**Table 2:** Mean CAPE and SF-12 scores in patients with RA, patients with SpA, and healthy controls.

	Rheumatoid Arthritis Mean (SD)	Spondyloarthritis Mean (SD)	Healthy Controls Mean (SD)	*p* Value	*p*-Value RA vs. Healthy	*p*-Value SpA vs. Healthy	*p*-Value RA vs. SpA
CAPE total frequency	1.68 (0.37)	1.65 (0.34)	1.49 (0.26)	<0.001	<0.001	0.002	0.563
CAPE total distress	2.40 (0.61)	2.28 (0.60)	2.03 (0.45)	<0.001	<0.001	0.009	0.173
Positive symptoms frequency	1.38 (0.27)	1.33 (0.25)	1.29 (0.22)	0.051	0.058	0.227	0.227
Negative symptoms frequency	1.90 (0.50)	1.88 (0.49)	1.63 (0.36)	<0.001	<0.001	0.001	0.694
Depressive symptoms frequency	2.07 (0.62)	2.04 (0.58)	1.73 (0.43)	<0.001	<0.001	<0.001	0.764
Positive symptoms distress	2.05 (0.59)	1.94 (0.58)	1.71 (0.44)	<0.001	<0.001	0.026	0.157
Negative symptoms distress	2.45 (0.68)	2.31 (0.69)	2.04 (0.50)	<0.001	<0.001	0.008	0.145
Depressive symptoms distress	2.66 (0.75)	2.54 (0.74)	2.29 (0.62)	0.002	0.001	0.053	0.206
SF12 Physical Component	32.58 (12.54)	30.02 (13.33)	53.31 (10.56)	<0.001	<0.001	<0.001	0.108
SF12 Mental Component	43.75 (11.15)	45.54 (10.50)	43.19 (9.55)	0.145	0.255	0.153	0.255

CAPE: Community Assessment of Psychic Experiences questionnaire; SpA: spondyloarthritis; RA: rheumatoid arthritis; SD: standard deviation.

**Table 3:** Factors independently associated with SF-12 scores.

	SF12 Physical Component		SF12 Mental Component	
	β Coefficient (SD)	*p*-Value	β Coefficient (SD)	*p*-Value
(Intercept)	66.33 (3.39)	<0.001	36.49 (2.79)	<0.001
Age	−0.17 (0.05)	0.002	0.20 (0.04)	<0.001
Gender (female)	−3.95 (1.75)	0.024	−0.61 (1.43)	0.670
SpA (disease-free ref.)	−19.70 (3.81)	<0.001	7.15 (3.13)	0.023
RA (disease-free ref.)	−15.83 (3.63)	<0.001	5.18 (2.98)	0.083
Marital status	0.73 (1.36)	0.592	1.79 (1.12)	0.110
Psychiatric history	−4.53 (1.40)	0.001	−5.17 (1.15)	<0.001
Smoking	−1.21 (1.60)	0.450	−4.24 (1.32)	0.001
Alcohol	0.45 (2.36)	0.850	2.08 (1.93)	0.282
History of drug use	2.43 (3.47)	0.485	−2.8 (2.85)	0.326

RA: rheumatoid arthritis; ref: reference; SD: standard deviation; SpA: spondyloarthritis.

## Data Availability

Data are available upon reasonable request.
